# Lenvatinib plus TACE with or without pembrolizumab for the treatment of initially unresectable hepatocellular carcinoma harbouring PD-L1 expression: a retrospective study

**DOI:** 10.1007/s00432-021-03767-4

**Published:** 2021-08-28

**Authors:** Song Chen, Zhiqiang Wu, Feng Shi, Qicong Mai, Liguang Wang, Fan Wang, Wenquan Zhuang, Xiaoming Chen, Huanwei Chen, Bo Xu, Jiaming Lai, Wenbo Guo

**Affiliations:** 1 Department of Interventional Radiology, The First Affiliated Hospital, Sun Yat-Sen University, No. 58, Zhongshan 2nd Road, Yuexiu District, Guangzhou, 510080 China; 2 Department of Interventional Radiology, Cancer Center, Guangdong Provincial People’s Hospital, Guangdong Provincial Academy of Medical Sciences, No. 106, Zhongshan 2nd Road, Yuexiu District, Guangzhou, 510080 China; 3 Department of Hepatic Surgery, Foshan First People’s Hospital, No. 81, North Lingnan Dadao, Chancheng District, Foshan, 528000 China; 4 Department of Thoracic Surgery, The First Affiliated Hospital, Sun Yat-Sen University, No. 58, Zhongshan 2nd Road, Yuexiu District, Guangzhou, 510080 China; 5 Department of Hepatobiliary-pancreatic Surgery, The First Affiliated Hospital, Sun Yat-Sen University, No. 58, Zhongshan 2nd Road, Yuexiu District, Guangzhou, 510080 China

**Keywords:** Pembrolizumab, Transarterial chemoembolization, Lenvatinib, Hepatectomy, Hepatocellular carcinoma, Conversion

## Abstract

**Purpose:**

The aim of this retrospective study was to compare the clinical outcomes of pembrolizumab-lenvatinib-transarterial chemoembolization (TACE) versus lenvatinib-TACE sequential therapy in selected populations of Chinese patients with initially unresectable hepatocellular carcinoma (uHCC) harbouring programmed cell death ligand-1 (PD-L1) expression.

**Methods:**

Consecutive patients with initial PD-L1-positive uHCC who received pembrolizumab-lenvatinib-TACE or lenvatinib-TACE sequential therapy were retrospectively identified from three medical institutions during 2016–2020. The primary endpoints included the rate of conversion therapy, defined as converting initially uHCC to hepatectomy, overall survival (OS), and progression-free survival (PFS); secondary endpoint was the frequency of key adverse events (AEs).

**Results:**

In total, 220 consecutively recruited patients were retrospectively reviewed, 78 of whom were ineligible according to the current criteria, leaving 142 patients [pembrolizumab-lenvatinib-TACE: *n* = 70, median age 58 years (range 36–69) and lenvatinib-TACE: *n* = 72, 57 years (35–68)] who were eligible for the study. The median duration of follow-up was 27 months [95% confidence interval (CI), 26.3–28.7 months]. At the last follow-up, the rate of conversion therapy was 25.7% in the pembrolizumab-lenvatinib-TACE group and 11.1% in the lenvatinib-TACE group (*p* = 0.025). The median OS was 18.1 months (95% CI 16.5–20.7) in the pembrolizumab-lenvatinib-TACE group versus 14.1 months (95% CI 12.2–16.9) in the lenvatinib-TACE group [hazard ratio (HR) 0.56, 95% CI 0.38–0.83; *p* = 0.004]. A distinct difference in the median PFS interval between the groups was detected [9.2 months (95% CI 7.1–10.4) in the pembrolizumab-lenvatinib-TACE group vs. 5.5 months (95% CI 3.9–6.6) in the lenvatinib-TACE group (HR 0.60; 95% CI 0.39–0.91; *p* = 0.006)]. The rates of the key AEs assessed, which were hypertension, nausea, and rash, were higher in the pembrolizumab-lenvatinib-TACE group than in the lenvatinib-TACE group (all *p* < 0.05).

**Conclusion:**

Among the selected populations of patients with initial PD-L1-positive uHCC, pembrolizumab-lenvatinib-TACE sequential therapy may have promising antitumour activity, with an acceptable conversion rate and a well-characterized safety profile.

## Introduction

Hepatocellular carcinoma (HCC) is the third most frequent type of cancer and is the leading histological form of primary liver cancer, accounting for 80–90% of cases, and the incidence is predicted to continue to rise (Llovet et al. 2020; Pinato et al. 2021). Most patients have an increased risk of unresectable hepatocellular carcinoma (uHCC) (Feun et al. 2019). Irrespective of tumour aetiology, in patients with uHCC for whom curative interventions tend to be infeasible, current front-line management for uHCC is limited to the six FDA-approved systemic interventions or locoregional interventions (Feun et al. 2019; Finn et al. 2020a), and the prognosis tends to be poor (Ikeda et al. 2018). Furthermore, the management landscape for patients with HCC, predominantly those with uHCC, is precipitously evolving as innovative, evidence-based approaches gradually become available (Kudo et al. 2020; Wu et al. 2020). Selecting patients for individual management modalities has been a key aspect of improving the outcomes of patients with uHCC; however, upcoming updates to the uHCC management approach have been instigated by the development of conversion therapy (Zhang et al. 2018). Conversion therapy, a downstaging therapy, is defined as converting the initial uHCC to resectable HCC if durable responses occur, with a reduction in the number or size of uHCC to within tolerable benchmarks (Gholam et al. 2019). Evidence from previous studies (Lau and Lai 2007; Zhang et al. 2018) regarding patients with uHCC demonstrated that the 5-year survival rate for hepatectomy after downstaging tumours varies from 24.9 to 57%, which is almost equivalent to that of primary hepatectomy (30–60%) (Conti et al. 2016; Livraghi et al. 2008). In other words, uHCC patients undergoing hepatectomy following conversion therapy have a similar 5-year survival rate to patients who initially undergo hepatectomy. The increasing prevalence of conversion therapy for uHCC may lead to it becoming be a preferred option (Finn et al. 2020a).

The alternative strategies for uHCC treatment are rapidly evolving, and so-called expanded criteria with various therapeutic modalities (i.e., transarterial chemoembolization [TACE], radiation therapy, surgery, and/or systemic therapies) have been developed (Verslype et al. 2012). Pembrolizumab, a potent and highly selective IgG4-kappa humanized monoclonal antibody designed to directly block the interaction between programmed cell death 1 (PD-1) and programmed death ligand 1 (PD-L1), has revealed promising activity in patients with PD-1-positive uHCC (Makker et al. 2019). However, whether combining pembrolizumab with lenvatinib plus TACE can yield a high rate of conversion therapy and improve survival in Chinese patients with initial PD-1-positive uHCC remains unclear. Furthermore, the available data on the effect of these combination therapies are lacking. Hence, we conducted a multicentre retrospective study to compare the clinical outcomes of pembrolizumab-lenvatinib-TACE versus lenvatinib-TACE sequential therapy in selected populations of Chinese patients.

## Materials and methods

### Data

From July 1, 2016, to July 31, 2020, consecutive patients with initial uHCC harbouring PD-L1 expression who were treated with pembrolizumab-lenvatinib-TACE or lenvatinib-TACE sequential therapy, for whom baseline data were accessible, were retrospectively reviewed at our tertiary medical institutions (The First Affiliated Hospital, Sun Yat-sen University; Guangdong Provincial People’s Hospital; Foshan First People's Hospital). uHCC was confirmed by two or more experienced hepatobiliary surgeons based on the National Comprehensive Cancer Network (NCCN) guidelines [mid- and advanced-stage HCC, or insufficient remnant liver volume following hepatectomy (< 40% for cirrhosis; < 30% for non-cirrhosis)] (Verslype et al. 2012; Zhu et al. 2021). The inclusion criteria comprised the following: histologically or cytologically confirmed HCC (except sarcomatoid and mixed cholangio-HCC tumours) or clinically confirmed HCC in accordance with the American Association for the Study of Liver Diseases criteria (Muratori et al. 2010); PD-L1 positivity defined as a combined positive score (CPS) > 1 (El-Khoueiry et al. 2017); TACE-eligible patients; albumin-bilirubin (ALBI) (grade 1–3); Barcelona Clinic Liver Cancer (BCLC) stage B or C HCC; Child–Pugh class A (score 5–6); good Eastern Cooperative Oncology Group performance status (ECOG-PS) of 0 or 1; at least 1 measurable target nodule by the modified Response Evaluation Criteria in Solid Tumours (mRECIST, version 1.1); and acceptable heart, kidney, and bone marrow function. The key exclusion criteria comprised the following: missing or unevaluable data; tumour burden exceeding 50% of the total liver volume; confirmed invasion of the bile duct; prior HCC-related treatment (i.e., TACE and/or radiotherapy, an agent targeting T cell costimulation or checkpoint pathways, blood-enhancing management, or surgery); TACE refractoriness or failure attributed to tumour progression or cancer-related complications (i.e., hepatic artery injury, hepatic abscess), or advanced HCC at the point of diagnosis or performance status deterioration; one-off TACE treatment or persistent portal vein thrombosis or portal vein invasion with Vp4 (Jiang et al. 2017); interruption or discontinuity of treatment, irrespective of treatment-related adverse events (AEs); malignant tumours from other organs diagnosed using the currently recommended imaging technique; symptomatic brain metastases; serious medical conditions (i.e., septicemia, acute respiratory failure, or cerebral stroke); and mental disturbance.

### Study design and treatment

A retrospective multicentre study was conducted in which eligible patients had undergone pembrolizumab-lenvatinib-TACE versus lenvatinib-TACE sequential therapy for uHCC harbouring PD-L1 expression. The decision to manage using pembrolizumab-lenvatinib-TACE or lenvatinib-TACE sequential therapy was made by two or more experienced hepatobiliary surgeons. Pembrolizumab was administered intravenously at 200 mg once every 3 weeks (Feun et al. 2019). Lenvatinib was administered orally at 8 mg/day, regardless of body weight (Finn et al. 2020a). These patients were subsequently treated using TACE, which was conducted by the same senior interventional physicians (BX and WG) as previously described (Fu et al. 2021). The tip of the catheter was inserted into the artery branches for tumour feeding according to tumour size, location, and arterial supply. Embolization was initially conducted using different diameter microspheres or drug-eluting beads, and the trunk was ultimately embolized with an absorbable gelatine sponge until the bleeding stopped. Pharmorubicin was used as the chemotherapy drug. The TACE process was repeated if the lesion reduction was less than 50% of the baseline. The TACE process was not repeated if patients underwent hepatectomy or if the tumour was determined to be Child–Pugh class C during the follow-up period. The criteria for selecting patients who can receive hepatectomy the same as those for primary hepatectomy: Child–Pugh class A, ALBI grade 1–2, a standard indocyanine green test at 15 min, an American Society of Anesthesiologists (ASA) physical status of 1–3. Drug treatment was suspended for 2–4 weeks after hepatectomy and continued until tumour progression, the onset of intolerable or serious AEs, or death. Antiviral therapy (tenofovir or sofosbuvir) was allowed in the study. The viral load was monitored during the follow-up period.

### Outcomes and assessments

The analysis of tumour PD-L1 expression was conducted by means of the PD-L1 IHC 22C3 pharmDx assay (Agilent Technologies, Carpinteria, CA) to assess the CPS, which was defined as the ratio of PD-L1-positive cells (tumour cells, lymphocytes, and macrophages) to the total number of viable tumour cells multiplied by 100 (Zhu et al. 2018). The primary endpoints were the rate of conversion therapy, OS, and PFS. The conversion rate was defined as the ratio of successfully converted uHCC individuals to total uHCC individuals. Tumour response and resectability were assessed according to contrast-enhanced computerized tomography (CT)/dynamic magnetic resonance imaging (MRI). OS was defined from the date of the initiation of combination therapy to the date death arising from any cause; PFS was defined from the date of the initiation of combination therapy to the date of progression as per the mRECIST version 1.1 or the date of death from any cause, whichever came first. Survival data for these patients undergoing multiple lines of therapy were collected regularly. The efficacy of TACE was assessed using dynamic CT/MRI after 1–2 cycles. TACE failure or refractory was defined based on the updated JSH criteria (Kudo 2015). The secondary endpoint was the frequency of key AEs, which were evaluated using the National Cancer Institute Common Terminology Criteria for Adverse Events, v 5.0. The collection of key AEs was conducted after the initiation of combination therapy. Follow-up occurred every 3 weeks to assess endpoint variables until tumour progression, onset of intolerable or serious AEs, or death. Therapeutic efficacy was assessed by CT and/or MR images, which was consistent with the follow-up schedule.

### Statistical analysis

Continuous data were compared using *t* tests or Mann–Whitney *U* tests. Categorical data were compared using Chi-squared tests. The cumulative survival rates after the initiation of combination therapy were estimated by the Kaplan–Meier method, followed by comparison using the log-rank test. Any survival-related variable with *p* < 0.10 in the univariate analysis was merged into a multivariable Cox proportional hazards model. HR was calculated using a Cox proportional hazards model, with the age, gender, cirrhosis, ALBI, Child–Pugh class A, BCLC stage B or stage C, alpha fetoprotein (AFP); ECOG-PS, HCC aetiology, PD-L1 expression, and number of metastatic sites used as covariates, and intervention provided as the time-dependent factor. The reverse Kaplan–Meier method was used to estimate the median follow-up time. A two-tailed *p* value < 0.05 was considered significant. All statistical analyses were conducted using SPSS 26.0 (IBM, Inc., New York) or GraphPad Prism 8.0 (La Jolla, California, United States).

## Results

### Demographic characteristics

**Fig. 1:**
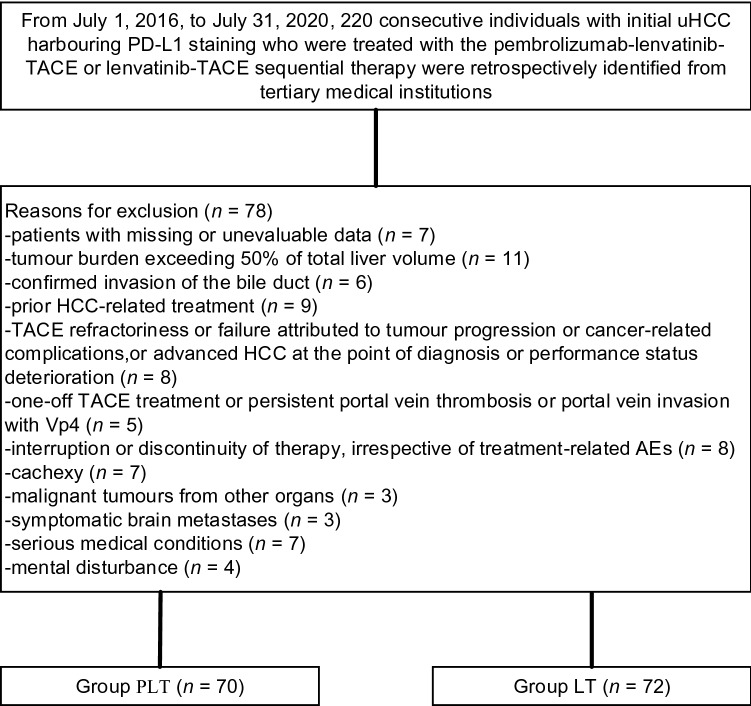
Flow diagram exhibiting the methods applied to identify objects to compare the clinical outcomes of the pembrolizumab-lenvatinib-transarterial chemoembolization (TACE) versus lenvatinib-TACE sequential therapy in selected populations of Chinese patients with initially unresectable hepatocellular carcinoma (uHCC) harbouring programmed cell death ligand-1 (PD-L1) staining

We identified 220 consecutive patients with uHCC harbouring PD-L1 expression who were treated with the pembrolizumab-lenvatinib-TACE or lenvatinib-TACE regimen, of whom 78 were eliminated according to the inclusion and exclusion criteria. Ultimately, a total of 142 patients were eligible, of whom 70 were treated with the pembrolizumab-lenvatinib-TACE regimen and 72 were treated with the lenvatinib-TACE regimen (Fig. 1). The demographics and baseline characteristics are shown in Table 1. The median age was 58 years (range 36–69) in the pembrolizumab-lenvatinib-TACE group and 57 years (range 35–68) in the lenvatinib-TACE group. At baseline, BCLC was stage B in 67.1% and stage C in 32.9% of patients undergoing pembrolizumab-lenvatinib-TACE versus stage B in 62.5% and stage C in 37.5% of patients undergoing lenvatinib-TACE (*p* = 0.564). ECOG-PS was 0 in 38.6% and 1 in 61.4% of patients receiving pembrolizumab-lenvatinib-TACE versus 0 in 41.7% and 1 in 58.3% of patients receiving lenvatinib-TACE (*p* = 0.708). The PD-L1 CPS was 1–20 in 45.7%, 20–50 in 34.3%, and 50–100 in 20.0% of patients who were treated with pembrolizumab-lenvatinib-TACE versus 1–20 in 50.0%, 20–50 in 29.2%, and 50–100 in 20.8% of patients who were treated with lenvatinib-TACE (*p* = 0.740). The baseline data were well-balanced between the groups. The median duration of treatment was 22 months (range 1–36) in the pembrolizumab-lenvatinib-TACE group and 22 months (range 1–36) in the lenvatinib-TACE group. The median number of treatment cycles was 26 (range 1–29) in the pembrolizumab-lenvatinib-TACE group and 27 (range 1–28) in the lenvatinib-TACE group.

**Table 1:** Baseline data of patients included in the study

Variable	PLT (*n* = 70)	LT (*n* = 72)	*p* value
Age (years), *n* (%)			0.611^a^
< 50	32 (45.7)	36 (50.0)	
≥ 50	38 (54.3)	36 (50.0)	
Gender, *n* (%)			0.992^b^
Male	37 (52.9)	38 (52.8)	
Female	33 (47.1)	34 (47.2)	
Cirrhosis, *n* (%)			0.758^b^
Absent	41 (58.6)	44 (61.1)	
Present	29 (41.4)	28 (38.9)	
ALBI grade, *n* (%)			0.514^b^
1	24 (34.3)	21 (29.2)	
2	46 (65.7)	51 (70.8)	
BCLC, *n* (%)			0.564^b^
Stage B	47 (67.1)	45 (62.5)	
Stage C	23 (32.9)	27 (37.5)	
AFP, *n* (%)			0.697^a^
< 400 ng/mL	25 (35.7)	28 (38.9)	
≥ 400 ng/mL	45 (64.3)	44 (61.1)	
ECOG-PS, *n* (%)			0.708^b^
0^**#**^	27 (38.6)	30 (41.7)	
1^**##**^	43 (61.4)	42 (58.3)	
Time since diagnosis, month (s)	5 (1–9)	5 (1–8)	0.317^a^
HCC aetiology, *n* (%)			0.361^b^
Hepatitis B virus	38 (54.3)	44 (61.1)	
Hepatitis C virus	21 (30.0)	20 (27.8)	
Without	11 (15.7)	8 (11.1)	
PD-L1 expression (CPS cut-off values)*, *n* (%)			0.740^b^
1–20	32 (45.7)	36 (50.0)	
20–50	24 (34.3)	21 (29.2)	
50–100	14 (20.0)	15 (20.8)	
Brain metastasis, *n* (%)			0.808^b^
Asymptomatic	48 (68.6)	52 (72.2)	
Without	22 (31.4)	20 (27.8)	
Duration of treatment month (s)	17 (1–34)	18 (1–34)	0.292^a^
Number of metastatic sites, *n* (%)			0.243^b^
< 3	29 (41.4)	32 (44.4)	
≥ 3	41 (58.6)	40 (55.6)	

*PLT* pembrolizumab-lenvatinib-transarterial chemoembolization, *LT* lenvatinib-transarterial chemoembolization, *ALBI* albumin–bilirubin, *BCLC* Barcelona clinic liver cancer, *AFP* alpha fetoprotein, *ECOG-PS* Eastern Collaborative Oncology Group performance status, *HCC* hepatocellular carcinoma, *PD-L1 *programmed cell death ligand-1, *CPS* combined positive score

*patients with high PD-L1 expression had a better outcome

^**#**^Fully active, able to carry on all pre-disease performance without restriction

^**##**^Restricted in physically strenuous activity but ambulatory and able to carry out work of a light or sedentary nature

^a^Mann–Whitney *U* test

^b^Independent samples *t* test

### Efficacy

**Fig. 2:**
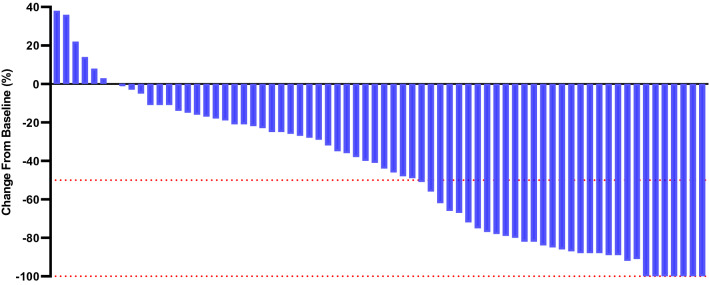
Percentage change from baseline in sums of diameters of target lesions by mRECIST version 1.1 in patients with initially unresectable hepatocellular carcinoma (uHCC) harbouring programmed cell death ligand-1 (PD-L1) staining who underwent the pembrolizumab-lenvatinib-transarterial chemoembolization (TACE) (*n* = 70)

**Fig. 3:**
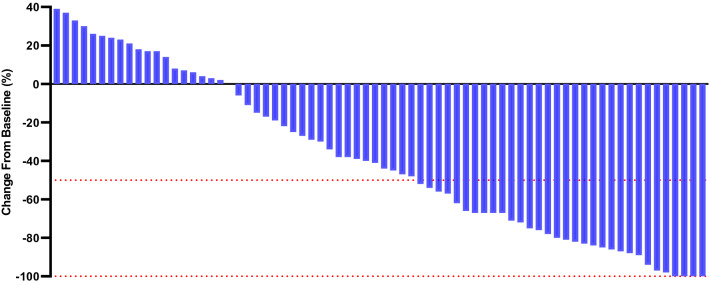
Percentage change from baseline in sums of diameters of target lesions by mRECIST version 1.1 in patients with initially unresectable hepatocellular carcinoma (uHCC) harbouring programmed cell death ligand-1 (PD-L1) staining who underwent the lenvatinib-transarterial chemoembolization (TACE) (*n* = 72)

**Fig. 4:**
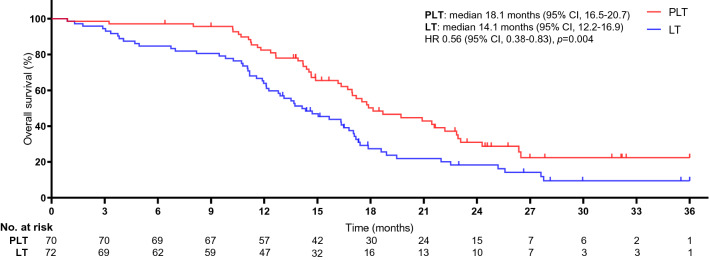
Kaplan–Meier curves for overall survival. The median overall survival was 18.1 months [95% confidence interval (CI) 16.5–20.7] for PLT and 14.1 months (95% CI 12.2–16.9) for LT (HR 0.56, 95% CI 0.38–0.83; *p* = 0.004). *The hazard ratio was calculated using a Cox proportional hazards model, with the age, gender, cirrhosis, ALBI, Child–Pugh class A, BCLC stage B or stage C, AFP; ECOG-PS, HCC aetiology, PD-L1 expression, and number of metastatic sites used as covariates, and intervention provided as the time-dependent factor

**Fig. 5:**
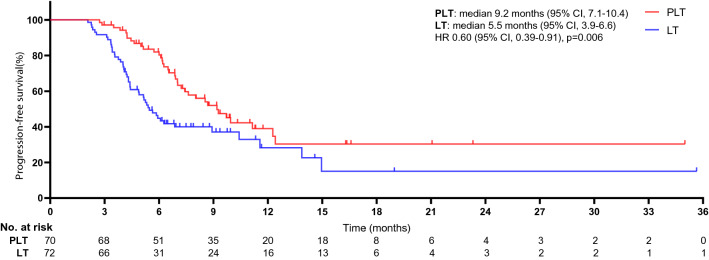
Kaplan–Meier curves for progression-free survival. The median progression-free survival was 9.2 months [95% confidence interval (CI) 7.1–10.4] for PLT and 5.5 months (95% CI 3.9–6.6) for LT (HR 0.60, 95% CI 0.39–0.91; *p* = 0.006). *The hazard ratio was calculated using a Cox proportional hazards model, with the age, gender, cirrhosis, ALBI, Child–Pugh class A, BCLC stage B or stage C, AFP; ECOG-PS, HCC aetiology, PD-L1 expression, and number of metastatic sites used as covariates, and intervention provided as the time-dependent factor

The study met the primary endpoints of the rate of conversion therapy, OS rate, and PFS rate. The median duration of follow-up was 27 months (95% CI 26.3–28.7 months). The objective response rate was 47.1% in the pembrolizumab-lenvatinib-TACE group vs. 27.8% in the lenvatinib-TACE group (*p* = 0.017). The disease control rate was 70.0% in the pembrolizumab-lenvatinib-TACE group vs. 52.8% in the lenvatinib-TACE group (*p* = 0.036). Significant difference was detected in reductions in tumour size per independent imaging review by mRECIST version 1.1 [90.0% (63 of 70) in the pembrolizumab-lenvatinib-TACE group vs. 72.2% (52 of 72) in the lenvatinib-TACE group, *p* = 0.007], as shown in Figs. 2 and 3. ALBI grade was 1 in 54.3% and 2 in 45.7% of patients experiencing pembrolizumab-lenvatinib-TACE versus 1 in 40.3% and 2 in 59.7% of patients experiencing lenvatinib-TACE (*p* = 0.030). A significant difference was seen regarding the number of patients who underwent hepatectomy (18 individuals in the pembrolizumab-lenvatinib-TACE group vs. 8 in the lenvatinib-TACE group). The rate of conversion therapy was 25.7% in the pembrolizumab-lenvatinib-TACE group and 11.1% in the lenvatinib-TACE group (*p* = 0.025) (Table 2). For patients who underwent hepatectomy, 4 (22.2%) died in the pembrolizumab-lenvatinib-TACE group and 6 (75.0%) died in the lenvatinib-TACE group (*p* = 0.012). For the entire study population, the 3-, 6-, and 12-month OS rates were 98.5%, 97.1%, and 82.4%, respectively, in the pembrolizumab-lenvatinib-TACE group and 94.4%, 84.7%, and 63.8%, respectively, in the lenvatinib-TACE group. A marked difference was noted in the median OS times between the groups [18.1 months (95% CI 16.5–20.7) in the pembrolizumab-lenvatinib-TACE group vs. 14.1 months (95% CI 12.2–16.9) in the lenvatinib-TACE group], as exhibited in Fig. 4. The pembrolizumab-lenvatinib-TACE regimen significantly improved the median OS time relative to that achieved with the lenvatinib-TACE regimen, and the triple therapy resulted in a marked 44% lower risk of death compared with the lenvatinib-TACE therapy (HR 0.56; 95% CI 0.38–0.83; *p* = 0.004). A remarkable distinction of 4.0 months in the median OS time was seen, and the pembrolizumab-lenvatinib-TACE regimen may be superior to the lenvatinib-TACE regimen, since the separation of both survival curves was maintained until the last follow-up. In addition, a distinct difference in the median PFS time between the groups was detected [9.2 months (95% CI 7.1–10.4) in the pembrolizumab-lenvatinib-TACE group vs. 5.5 months (95% CI 3.9–6.6) in the lenvatinib-TACE group (HR 0.60; 95% CI 0.39–0.91; *p* = 0.006)], as presented in Fig. 5.

**Table 2:** Therapeutic efficacy of response and conversion therapy

Variable	PLT (*n* = 70)	LT (*n* = 72)	*p* value^a^
Best overall response, *n* (%)			0.016
CR	7 (10.0)	4 (5.6)	
PR	26 (37.1)	16 (22.2)	
SD	16 (22.9)	18 (25.0)	
PD	18 (25.7)	30 (41.7)	
Unidentified	3 (4.3)	4 (5.6)	
Objective response rate, *n* (%)	33 (47.1)	20 (27.8)	0.017
Disease control rate, *n* (%)	49 (70.0)	38 (52.8)	0.036
Conversion therapy, *n* (%)			0.025
Hepatectomy	18 (25.7)	8 (11.1)	
Without hepatectomy	52 (74.3)	64 (88.9)	

*PLT* pembrolizumab-lenvatinib-transarterial chemoembolization, *LT* lenvatinib-transarterial chemoembolization, *CR* complete response, *PR* partial response, *SD* stable disease, *PD* progressive disease

^a^Mann–Whitney *U* test

A subgroup analysis was conducted according to the CPS scores. For PD-L1 CPS ≥ 50, the median OS time was 19.5 months in the pembrolizumab-lenvatinib-TACE group and 15.7 months in the lenvatinib-TACE group (HR 0.34; *p* = 0.001). For patients with PD-L1 CPS ≥ 20, the median OS time was 18.3 months in the pembrolizumab-lenvatinib-TACE group and 14.6 months in the lenvatinib-TACE group (HR 0.54; *p* = 0.007). For patients with PD-L1 CPS > 1, the median OS time was 15.1 months in the pembrolizumab-lenvatinib-TACE group and 12.4 months in the lenvatinib-TACE group (HR 0.35, *p* = 0.003). A higher PD-L1 CPS was associated with increased survival benefits with anti-PD-L1 treatment.

### Safety

A total of 108 patients (76.1%) suffered one or more AEs. Table 3 summarizes the key AEs observed. Of the 142 patients, treatment discontinuation occurred in 16 (11.3%) patients and this discontinuation was primarily attributed to treatment-related AEs (9 of 70 patients in the pembrolizumab-lenvatinib-TACE group and 7 of 72 in the lenvatinib-TACE group, *p* = 0.556). Thirteen patients in the pembrolizumab-lenvatinib-TACE group and 2 in the pembrolizumab-lenvatinib group experienced hypertension (*p* = 0.002). In addition, between-group significant differences were also seen in terms of nausea (15.7% in the pembrolizumab-lenvatinib-TACE group vs. 4.1% in the lenvatinib-TACE group, *p* = 0.021) and rash (12.9% in the pembrolizumab-lenvatinib-TACE group vs. 2.8% in the lenvatinib-TACE group, *p* = 0.004). There were no significant differences in other grade ≥ 3 AEs. The key grade ≥ 3 AEs observed were elevated AST, elevated ALT, thrombocytopaenia, hypertension, fatigue, asthenia, and rash, which were generally manageable. No patients in either group experienced treatment-related death.

**Table 3:** Key treatment-related adverse events of ≥ grade 3

Variable *n* %	PLT (*n* = 70)	LT (*n* = 72)	HR (95%)	*p* value^a^
Elevated AST	17 (24.3)	11 (15.7)	1.17 (0.19–2.53)	0.179
Elevated ALT	16 (22.9)	12 (17.1)	3.25 (0.76–4.81)	0.356
Thrombocytopaenia	5 (7.1)	3 (4.1)	2.58 (0.38–3.35)	0.444
Hypertension	13 (18.6)	2 (2.8)	2.32 (1.43–3.54)	0.002
Fatigue	7 (10.0)	2 (2.8)	1.69 (0.21–2.22)	0.078
Asthenia	6 (8.6)	4 (5.6)	3.11 (0.67–4.35)	0.338
Fever	4 (5.7)	5 (6.9)	3.36 (0.39–4.53)	0.764
Nausea	11 (15.7)	3 (4.1)	2.70 (1.73–3.21)	0.021
Hypothyroidism	4 (5.7)	1 (1.4)	4.76 (0.85–5.35)	0.164
Arthralgia	2 (2.9)	0 (0.0)	1.12 (0.16–2.54)	0.150
Decreased appetite	5 (7.1)	4 (5.6)	2.34 (0.42–3.22)	0.699
Diarrhea	5 (7.1)	3 (4.1)	4.12 (0.86–4.35)	0.444
Rash	12 (12.9)	2 (2.8)	1.67 (1.07–2.54)	0.004
Pruritus	2 (2.9)	1 (1.4)	3.25 (0.84–4.22)	0.544

*PLT* pembrolizumab-lenvatinib-transarterial chemoembolization, *LT* lenvatinib-transarterial chemoembolization, *AST* aspartate aminotransferase, *ALT* alanine aminotransferase, *HR* hazard ratio

^a^Mann–Whitney *U *test;

## Discussion

The findings of the retrospective review demonstrated that the pembrolizumab-lenvatinib-TACE regimen may contribute to a higher rate of conversion therapy and longer survival time in selected populations of Chinese individuals with initial PD-L1-positive uHCC than the lenvatinib-TACE regimen, without unexpected safety-related complications. The high rate of conversion therapy suggested that the triple therapy may significantly preclude the progression of uHCC harbouring PD-L1 expression. The early-stage survival curves among individuals undergoing the triple therapy continued until the last follow-up date, with a distinct 4.0-month distinction in median OS times between the two groups.

The results of this retrospective study are in accordance with the findings of the phase 3 KEYNOTE-240 trial (Kudo et al. 2020) involving the assessment of the efficacy and safety of pembrolizumab in 157 Asian individuals. The results showed that the median OS times [13.8 months (95% CI 10.1–16.9) vs. 8.3 months (95% CI 6.3–11.8), respectively; HR 0.55; 95% CI 0.37–0.80] and median PFS times [2.8 months (95% CI 2.6–4.1) vs. 1.4 months (95% CI 1.4–2.4), respectively; HR 0.48; 95% CI 0.32–0.70] were higher in the pembrolizumab group than in the placebo group. Their study confirmed the superiority of pembrolizumab anticancer activity compared with that achieved with the placebo. Furthermore, a trend towards superior survival benefit among patients treated with pembrolizumab was detected when compared with the survival rate of the entire population. Similarly, a nonrandomized, multicentre, open-label, phase 2 trial (Zhu et al. 2018) of 104 eligible patients with advanced HCC who were treated with pembrolizumab reported a median OS time of 12.9 months. Recently, a phase 3 trial (Finn et al. 2019) of pembrolizumab monotherapy demonstrated a median OS time of 13.9 months in patients with advanced HCC. However, a previous phase Ib single-arm study (Finn et al. 2020a) of 100 individuals treated with lenvatinib plus PD-1 inhibition with pembrolizumab showed improved anticancer activity, with a median OS time of 22.0 months. Although the definite cause driving the higher OS rate is unknown, the immunomodulatory effect of lenvatinib complements the pembrolizumab anticancer effect, thereby expanding the sensitivity of tumours to lenvatinib plus pembrolizumab combination therapy (Finn et al. 2020a; Finn et al. 2020c). Similar results have been reported in other agent targeting T-cell costimulation studies (El-Khoueiry et al. 2017; Finn et al. 2020b; Yau et al. 2020) involving uHCC.

In the present study, the triple therapy tended to provide a high conversion rate and improved the survival time of patients with initial uHCC harbouring PD-L1 expression. PD-L1-positive expression has previously been demonstrated to be associated with a high conversion rate and improved survival rate (Zhang et al. 2019; Zhu et al. 2018). PD-1/PD-L1 pathway inhibitory signals negatively regulate the immune response and may be one of the mechanisms of immune escape in HCC (El-Khoueiry et al. 2017; Feun et al. 2019). The lenvatinib-TACE regimen might increase the anticancer effect of pembrolizumab (Makker et al. 2019), which has been proposed to occur via immune microenvironment modulation (Wu et al. 2020). According to this assertion, the promising synergistic anticancer effect of the pembrolizumab-lenvatinib-TACE combination may primarily contribute to the efficacy of pembrolizumab, which enhances the anticancer activity of T cells (Ikeda et al. 2018; Makker et al. 2019).

The combination of lenvatinib with TACE has been demonstrated to remarkably prolong the OS time of patients with uHCC, leading to a significant advance in the management of uHCC (Fu et al. 2021; Kawamura et al. 2020). Although the mechanisms of resistance to lenvatinib-TACE are still unknown (Shimose et al. 2020), an explanation as to why a better survival benefit is observed with pembrolizumab is that lenvatinib-TACE forms a beneficial tumour-immune microenvironment, partially through blocking immunosuppressive vascular endothelial growth factor (VEGF) signalling (Kawamura et al. 2020); thus, the earlier pembrolizumab is applied, the greater the survival benefit may be for these individuals (Llovet et al. 2020; Zhu et al. 2018).

Prior studies (Han et al. 2020; Sieghart et al. 2013) have shown the anticancer activity of TACE in intrahepatic tumours, which might be ascribed to the local anticancer effect of TACE. Moreover, numerous clinical practice guidelines (Park et al. 2019; Yoon et al. 2018) have recommended the utilization of TACE as the standard of care for individuals with HCC (BCLC stage B). Undeniably, TACE can exacerbate hypoxia in tumour cells and trigger the upregulation of hypoxia-related factors (Han et al. 2020; Lewandowski et al. 2009). Increased expression of hypoxia-related factors, in turn, leads to the upregulation of VEGF and fibroblast growth factor and ultimately promotes tumour angiogenesis and progression, implying that blockade of VEGF receptors may have an anticancer effect (Luo et al. 2011; Pawlik et al. 2011). Lenvatinib, a receptor tyrosine kinase inhibitor, inhibits the kinase activities of VEGF receptors (targeting VEGFR-1,2,3) and other pathways and may overpower the upregulation of proangiogenic factors following TACE, which has been approved as an alternative front-line management for uHCC (Finn et al. 2020a; Fu et al. 2021). The combination of lenvatinib with TACE may block pathogenic angiogenesis and tumour growth (Kawamura et al. 2020; Shimose et al. 2020). Previous studies (Kawamura et al. 2020; Shimose et al. 2020) have provided clinical evidence that lenvatinib combined with TACE could provide survival benefits over TACE alone for uHCC. For extrahepatic tumours, the therapeutic effect of lenvatinib plus TACE is reduced, which may be attributed to an established mechanism to escape recognition by T cells (Fu et al. 2021; Ikeda et al. 2018). The mechanism for this finding may be associated with the immune evasion capacity of cancer cells (Kudo et al. 2020; Verslype et al. 2012). This might be the reason why uHCC can evade traditional treatment strategies (Verslype et al. 2012; Zhang et al. 2018). Patients with intrahepatic tumours receiving lenvatinib plus TACE have a distinct, durable response, suggesting that these patients derive short-term anticancer benefits from lenvatinib plus TACE (Fu et al. 2021; Kawamura et al. 2020).

Several limitations should be acknowledged. First, the retrospective nature of the current study has inherent shortcomings. Conversion rates and survival outcomes may be limited by the relatively small sample size and by selection biases, which may impede the ability to draw reliable conclusions regarding the comprehensive safety or efficacy of both regimens. The lack of uniform diagnostic procedures might have led to an overestimation of the conversion rates and survival outcomes. In view of median survival times and the risks of rapid progression, survival estimates in the study might have been, to some extent, restrained by the treatment timing. Patients with mental disorders, suicidal tendencies, and tumour burden exceeding 50% of the total liver volume may have influenced the outcome of the analysis. Aetiological analysis of the deaths was not detailed, which may also have led to an overestimation of the survival curves. Second, the PD­L1 status analysis was inconsistent, and comprehensive assessments of primary or acquired resistance to single PD-1 pathway blockade were not possible. Third, uniform consensus was lacking regarding the definition of surgical indications. Fourth, other outcomes (i.e., quality of life, potential risk factors for death) were not analyzed in this study.

## Conclusion

The results described here may support the increasing body of evidence that suggests that the pembrolizumab-lenvatinib-TACE combination contributes to a higher rate of conversion and more promising survival outcomes in selected populations of Chinese patients with initial uHCC harbouring PD-L1 expression than the lenvatinib-TACE combination, with a manageable safety profile. Nonetheless, due to the retrospective nature of the current study, we cannot make a definitive conclusion on the use of pembrolizumab-lenvatinib-TACE for the treatment of initial uHCC harbouring PD-L1 expression. It is important to consider the findings of the current study in the context of the overall uHCC management landscape.

## Data Availability

All data generated or analyzed during this study are included in the article.

## Abbreviations

TACE: Transarterial chemoembolization
uHCC: Unresectable hepatocellular carcinoma
PD-L1: Programmed cell death ligand-1
OS: Overall survival
PFS: Progression-free survival
AEs: Adverse events
CI: Confidence interval
HR: Hazard ratio
PD-1: Programmed cell death 1
NCCN: National comprehensive cancer network
CPS: Combined positive score
BCLC: Barcelona clinic liver cancer
ECOG-PS: Eastern Cooperative Oncology Group performance status
mRECIST: Modified response evaluation criteria in solid tumours
CT/MRI: Computerized tomography/dynamic magnetic resonance imaging
